# Supplementary figures and images for: Non-O1/O139 environmental Vibrio cholerae from Northern Cameroon reveals potential intra-/inter-continental transmissions
Source: PLoS Negl Trop Dis. 2025 Apr 3;19(4):e0012890. doi: 10.1371/journal.pntd.0012890 (PMC12037076; doi:10.1371/journal.pntd.0012890)

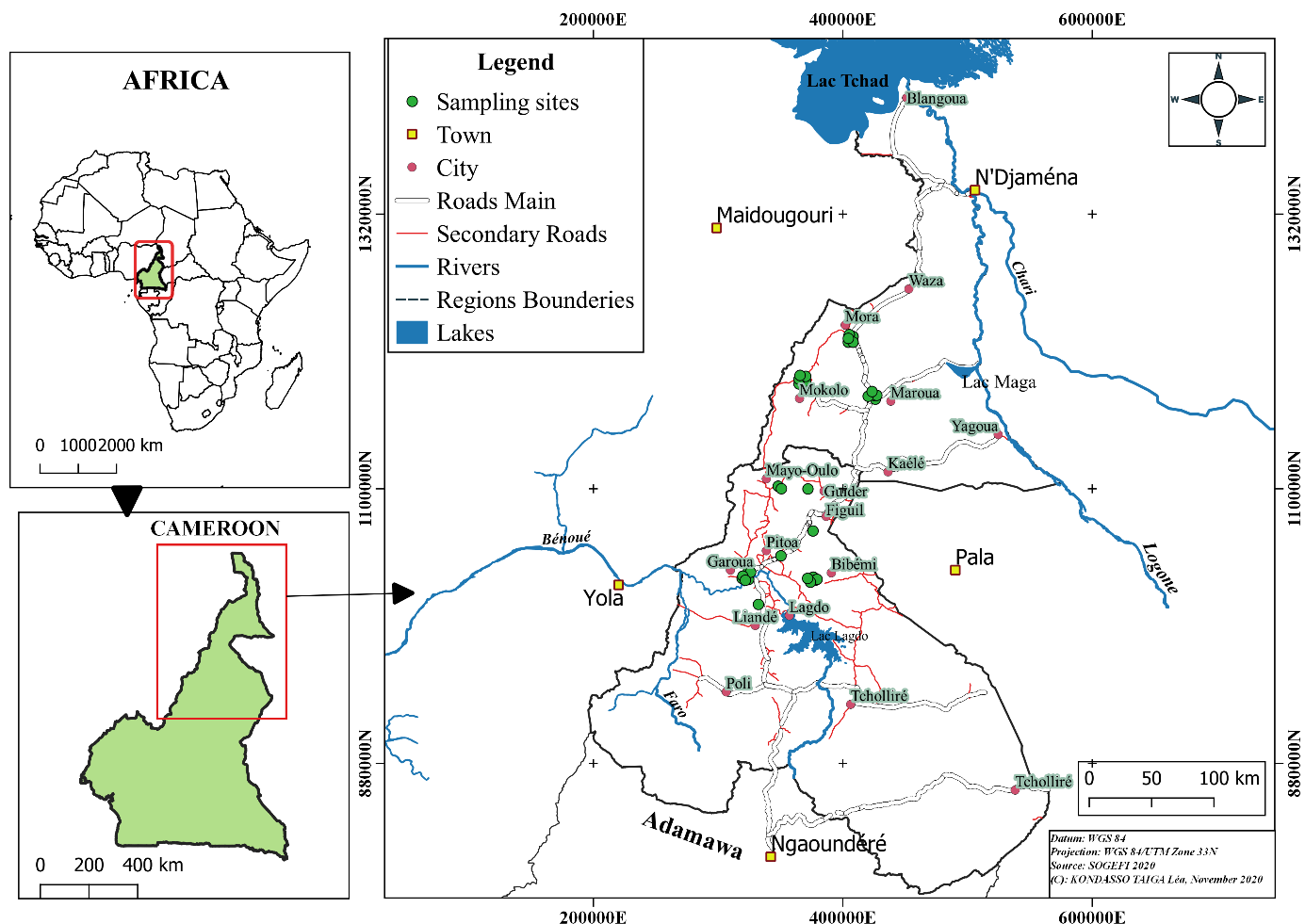

Supplement: S1 Fig — The base map was adapted from Diva-Gis (https://diva-gis.org/data.html). The license information is available at https://en.wikipedia.org/wiki/DIVA-GIS. (TIF) [file pntd.0012890.s001.tif]

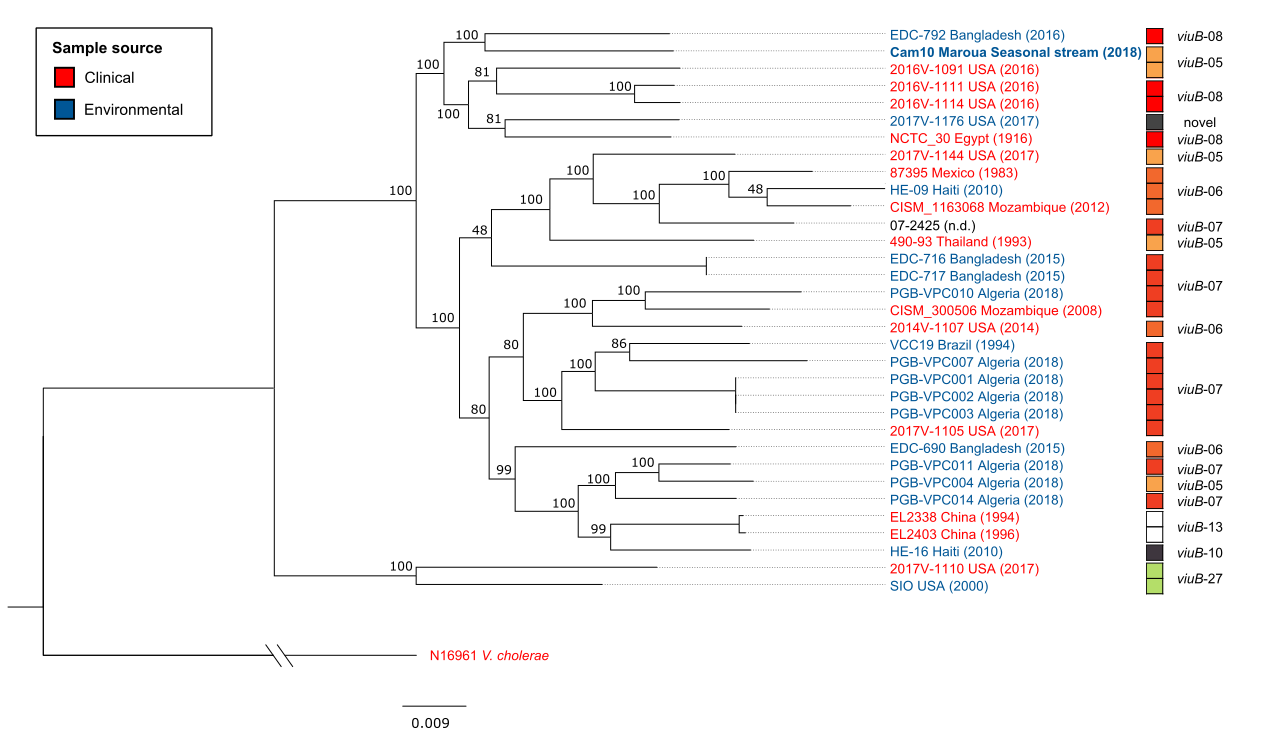

Supplement: S2 Fig — Maximum-likelihood (ML) tree was generated by RAxML with GTR+G model, using 2,323 core genes. viuB genotype of each strain is indicated on the right side of the tree. Each strain is color-coded by sample source, with clinical strains in red, environmental strains in blue, and one strain (07-2425) from unknown source in black. Strain Cam10 from this study is highlighted in bold. The tree is rooted with V. cholerae strain N16961 as an outgroup. Nodes are labeled with bootstrap support from 1,000 replicates. The scale bar represents the number of nucleotide substitutions per site. n.d.: no date (collection date unknown). (TIF) [file pntd.0012890.s002.tif]

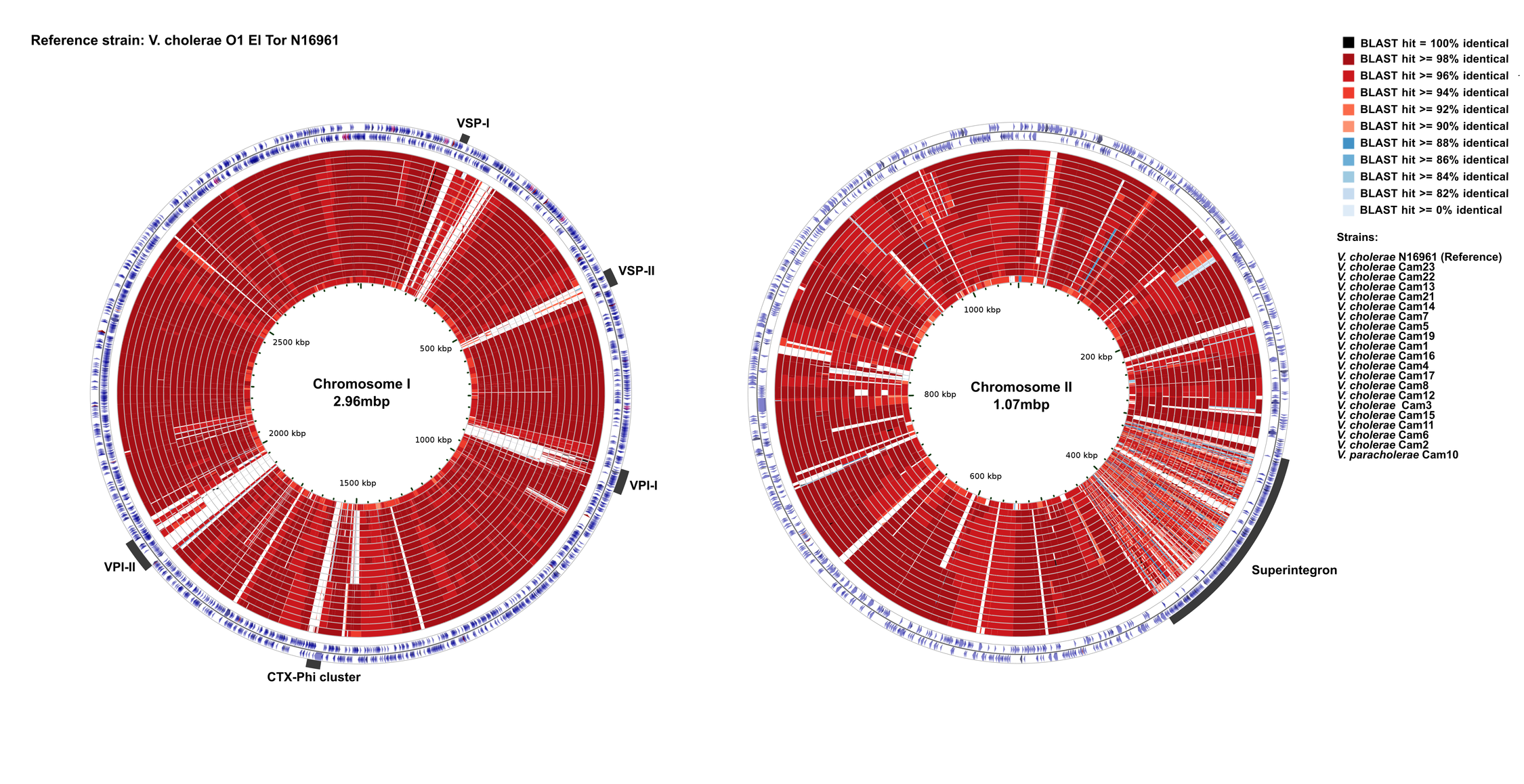

Supplement: S3 Fig — Genetic similarities across two chromosomes of V. cholerae and V. paracholerae strains are shown. The reference N16961 strain is depicted as the blue backbone, with forward and reverse strands separately. Gene regions with BLAST hits are colored according to the identity scores, while the blank regions indicate no hits. Primary pathogenicity islands and superintegron in N16961 are annotated around the circles. Strains are arranged from V. cholerae Cam23 on the outer circle to V. paracholerae Cam10 in the innermost circle, following the legend at the right of the plot. (TIF) [file pntd.0012890.s003.tif]

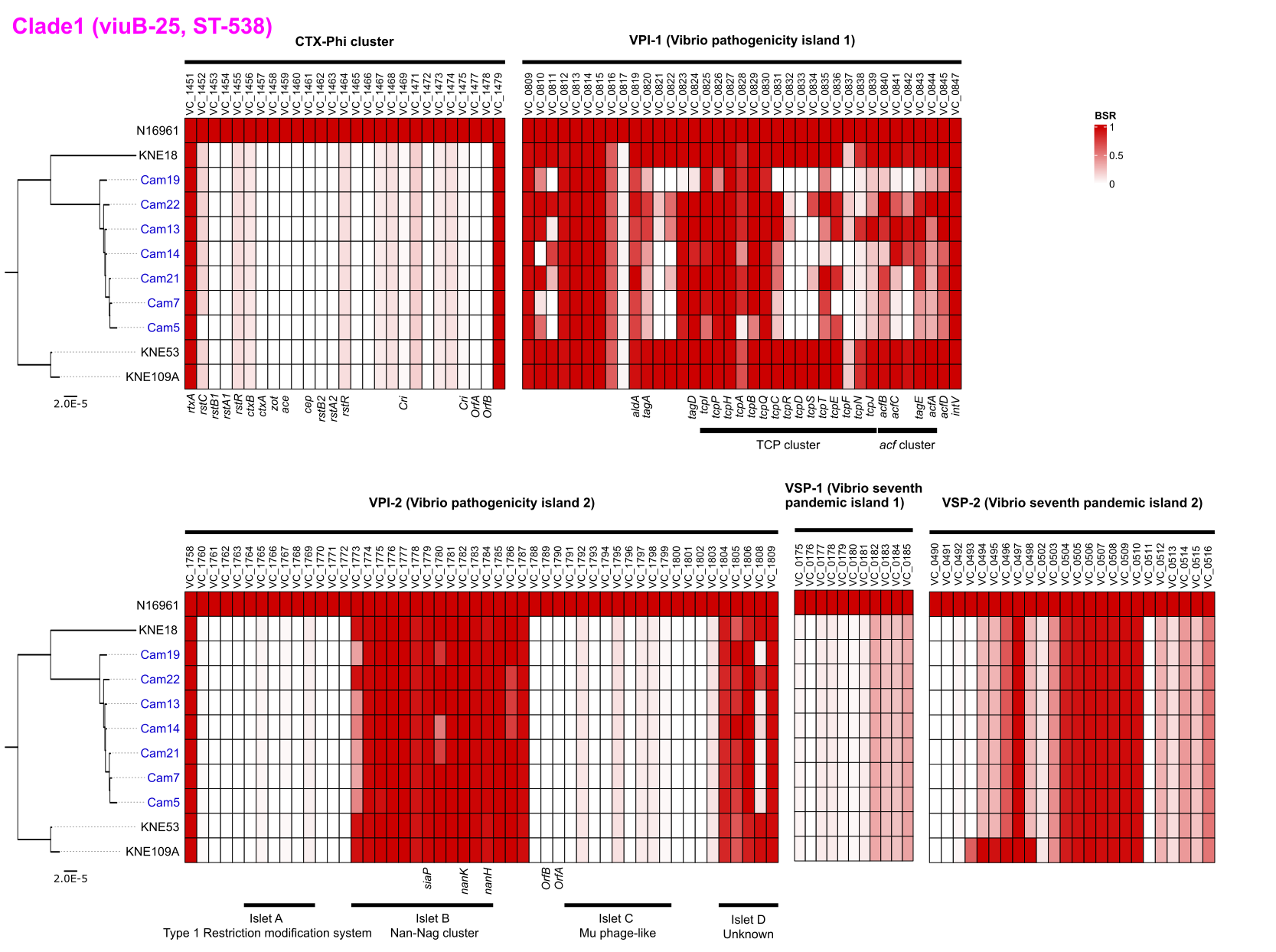

Supplement: S4 Fig — For each gene, presence and similarity are indicated by a color gradient from white (absent) to dark red (identical), according to the blast score ratio (BSR). Each row represents a different strain, with V. cholerae El Tor strain N16961 at the top as a reference. The strains from this study are highlighted in blue. (TIF) [file pntd.0012890.s004.tif]

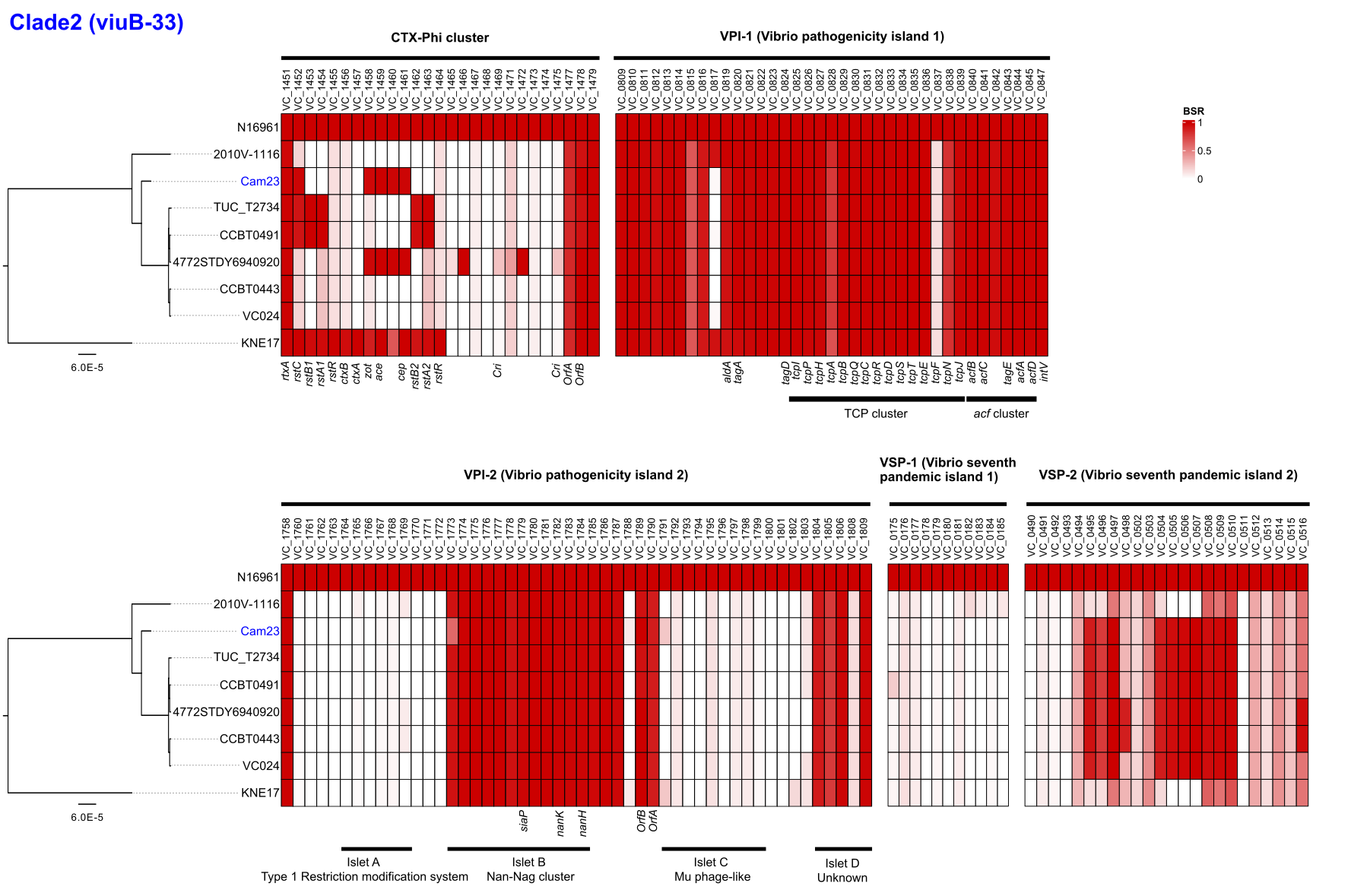

Supplement: S5 Fig — For each gene, presence and similarity are indicated by a color gradient from white (absent) to dark red (identical), according to the blast score ratio (BSR). Each row represents a different strain, with V. cholerae El Tor strain N16961 at the top as a reference. The strains from this study are highlighted in blue. (TIF) [file pntd.0012890.s005.tif]
