# Supplementary material for: Non-O1/O139 environmental Vibrio cholerae from Northern Cameroon reveals potential intra-/inter-continental transmissions
Source: PLoS Negl Trop Dis. 2025 Apr 3;19(4):e0012890. doi: 10.1371/journal.pntd.0012890 (PMC12037076; doi:10.1371/journal.pntd.0012890)
Supplement: Attachment — Submitted filename: Response to Reviewer1_v2.docx [file pntd.0012890.s011.docx]

**Manuscript Number: PNTD-D-24-01435**

**Response to Reviewers**

Dear Reviewer,

Thank you for giving us an opportunity to submit a revised version of our submission **“Non-O1/O139 environmental Vibrio cholerae from Northern Cameroon reveals potential intra-/inter-continental transmissions”** for publication as a research paper in **PLOS Neglected Tropical Diseases**. We sincerely appreciate the time and effort you dedicated to reviewing our manuscript, as well as all the insightful comments you provided.

We have carefully addressed your feedback in our revised submission. All changes have been tracked within the manuscript.

Please see below for detailed responses to each of the comments. Our responses for each comment are highlighted in blue.

### **Reviewer’s comments to the authors**

**Reviewer 1, Comment 1:**

Overall, the manuscript is very well written and structured. Therefore, I only have minor corrections to suggest:

- Page 5: „Water sample collection and isolation of V. cholerae“ – Please specify in this section how much water was filtered for a sample.
- Page 6: “DNA extraction & sequencing“ last sentence in the first paragraph - Please do not start a sentence with an abbreviated gene name. Please check this for the entire manuscript.
- Page 6: “DNA extraction & sequencing“ second paragraph - Please explain why were the two batches analyzed at different laboratories with different sequencing kits?
- Page 7: „Genome assembly and species identification“ - Please explain using identities in % what corresponds to a perfect match of viuB to the reference gene and when it is a mismatch.
- Page 12: „ of tcpA+ non-O1, non-O139 V. cholerae (NOVCs) and V. paracholerae from freshwater sources in Cameroon“ - Please always use lowercase letters for names, as ompU begins with a capital letter in the text on the start of page 12.

**Response:** Thank you for your suggestions. We have corrected the format and added other necessary information as follows:

- The volume of water filtered per sample (300mL) has been specified (Page 6).
- We have corrected sentences starting with abbreviated gene names throughout the manuscript.
- We have added why the samples were sequenced at different locations (because samples were collected in different years and new researchers from Singapore contributed to the sample analysis in the second year of the project with regards to their expertise.) (Page 6).
- We have included additional information on *viuB* genotyping under the section "Genome assembly and species identification" on Page 7. Out of the 816bp *viuB* gene, 272bp variable region was used for genotyping. The gene was considered as the same genotype if all nucleotides were identical (perfect match) or if there was only a single nucleotide mismatch within the 272bp region compared to the reference database.
- Capitalization of gene names has been standardized.

**Reviewer 1, Comment 2:**

Dear Authors,

Thank you for this detailed genetic comparison between O1/O139 *V. cholerae* and NOVC.

The authors discuss possible links between the association between O1/O139 *V. cholerae* and NOVC and their role in cholera transmission. In addition, the genetic virulence marker *tcpA* in NOVC, which shows similarities to that of classical O1 strains, suggests that these strains may play an important role in outbreak events and transmission. In addition, the authors found that non-O1/O139 *V. cholerae* strains from water sources in northern Cameroon are related to strains from Kenya and Argentina despite their genetic variability, suggesting cross-regional transmission. All these findings emphasize the need to consider non-O1/O139 strains in cholera surveillance, which may play a greater role in the spread of cholera than previously thought.

**Response:** Thank you for your positive feedback. We appreciate your comment on the relevance of non-O1/O139 strains in cholera research and surveillance to better understand their role in transmission dynamics.

Thank you once again for your reviews which have been invaluable in improving our manuscript.

Best regards,

Deborah Yebon Kang
